# Light Intensity Enhances the Lutein Production in *Chromochloris zofingiensis* Mutant LUT-4

**DOI:** 10.3390/md22070306

**Published:** 2024-06-29

**Authors:** Qiaohong Chen, Mingmeng Liu, Wujuan Mi, Dong Wan, Gaofei Song, Weichao Huang, Yonghong Bi

**Affiliations:** 1 State Key Laboratory of Freshwater Ecology and Biotechnology, Institute of Hydrobiology, Chinese Academy of Sciences, Wuhan 430072, China; qiaohongchen@ihb.ac.cn (Q.C.); miwj@ihb.ac.cn (W.M.); wangdong@ihb.ac.cn (D.W.); song@ihb.ac.cn (G.S.); huangwc@ihb.ac.cn (W.H.); 2 School of Civil Engineering, Hubei Engineering University, Xiaogan 432000, China; lmm007@hbeu.edu.cn

**Keywords:** *Chromochloris zofingiensis*, biomass, lutein, light intensity, organic carbon availability

## Abstract

*Chromochloris zofingiensis*, a unicellular green alga, is a potential source of natural carotenoids. In this study, the mutant LUT-4 was acquired from the chemical mutagenesis pool of *C. zofingiensis* strain. The biomass yield and lutein content of LUT-4 reached 9.23 g·L^−1^, and 0.209% of dry weight (DW) on Day 3, which was 49.4%, and 33% higher than that of wild-type (WT), respectively. The biomass yields of LUT-4 under 100, 300, and 500 µmol/m^2^/s reached 8.4 g·L^−1^, 7.75 g·L^−1^, and 6.6 g·L^−1^, which was 10.4%, 21%, and 29.6% lower compared with the control, respectively. Under mixotrophic conditions, the lutein yields were significantly higher than that obtained in the control. The light intensity of 300 µmol/m^2^/s was optimal for lutein biosynthesis and the content of lutein reached 0.294% of DW on Day 3, which was 40.7% more than that of the control. When LUT-4 was grown under 300 µmol/m^2^/s, a significant increase in expression of genes implicated in lutein biosynthesis, including phytoene synthase (*PSY*), phytoene desaturase (*PDS*), and lycopene epsilon cyclase (*LCYe*) was observed. The changes in biochemical composition, Ace-CoA, pyruvate, isopentenyl pyrophosphate (IPP), and geranylgeranyl diphosphate (GGPP) contents during lutein biosynthesis were caused by utilization of organic carbon. It was thereby concluded that 300 µmol/m^2^/s was the optimal culture light intensity for the mutant LUT-4 to synthesize lutein. The results would be helpful for the large-scale production of lutein.

## 1. Introduction

Lutein, a naturally occurring carotenoid, has garnered significant attention due to its potential health benefits, including the scavenging of free radicals, prevention or amelioration of cardiovascular diseases, age-related macular degeneration (AMD), Alzheimer’s Disease (AD), and certain forms of cancer, as well as its advantageous effects on skin health [1,2]. Currently, the primary source for the commercial production of lutein is marigold petals [3]. In addition to requiring considerable effort to separate the petals and extract lutein from the marigold flowers, which comprise a meager 0.03% of dry weight (DW), the cultivation of the marigold plant is a labor-intensive undertaking [4]. Although lutein is frequently present in vegetables, not all populations receive enough of it on a daily basis. Thus, it is important to look for high-quality lutein sources for dietary supplements.

Microalgae are abundant sources of carotenoids, which can operate as primary carotenoids during photosynthesis or as secondary carotenoids in reaction to unfavorable conditions [5]. As an essential pigment for photosynthetic processes, microalgae lutein production is correlated with photosynthetic activity. In contrast to terrestrial plants, microalgae exhibit superior rates of growth and photosynthetic efficiency [6]. Some microalgal species, including *Chlorella protothecoides* [7], *Dunaliella salina* [8], *Muriellopsis* sp. [4], *Parachlorella* sp. JD-076 [9], *Scenedesmus obliquus* FSP-3 [4], and *Chlorella vulgaris* UTEX 265 [10], have been studied for lutein production, with limited progress.

Since photosynthetic pigment synthesis is a physiological response of microalgae cells to high light stress, photoautotrophic cultivation is frequently used in the production of lutein [11]. However, the dry weight in photoautotrophic microalgal cells was lower than that of those cultured heterotrophically [12]. Currently, mixotrophic culture integrates the benefits of autotrophy and heterotrophy [13]. Microalgae engaged in mixotrophic culture take up both organic and inorganic nutrients in the presence of light under conditions of aerobic respiration and photosynthesis [14]. Mixotrophic cultivation of microalgae has been shown to have numerous advantages, including a reduction in the photo-inhibitory effect of photosynthetic capacity, biomass loss at night, and photo-oxidative damage during the cultivation period [15]. In addition, light intensity is also an important parameter that has influenced the production of lutein in microalgae. When *Scenedesmus obliquus* FSP-3 was exposed to white light instead of blue, green, or red light, high lutein production was seen at a light intensity of 300 µmol/m^2^/s [4]. The growth of *Coccomyxa onubensis* under white light with an intensity of 400 µmol/m^2^/s led to a notable lutein production [16]. Thus, the generation of lutein from microalgae with high biomass and lutein content is now possible via mixotrophic culturing.

*Chromochloris zofingiensis* is a Chlorophyceae class green microalga that exhibits rapid growth under three different trophic modes (i.e., autotrophy, heterotrophy, and mixotrophy) [17]. Chlorophyll degradation and the accumulation of secondary carotenoids occurred when *C. zofingiensis* ceased photosynthesis in the presence of glucose [18]. The mutant CZ-LZM3 of *C. zofingiensis* strain has described a deficiency in astaxanthin accumulation but, on the other hand, accumulated significant quantities of three distinct carotenoids (namely lutein, zeaxanthin, and β-carotene) during heterotrophic cultivation [8]. Nevertheless, the contents of lutein in this mutant (i.e., CZ-LZM3) decreased significantly during heterotrophic growth. Therefore, it is essential to exert significant efforts to augment lutein production in *C. zofingiensis*.

This study investigated the biological profiles of *C. zofingiensis* mutant LUT-4 under different light intensities by linking the physiological properties and molecular characteristics to evaluate the potential of LUT-4 to produce lutein. The growth characteristics of mutant LUT-4 under different light intensities were determined. The main organic composition (i.e., lipid, protein, and carbohydrate) of mutant LUT-4 under optimal light intensity was measured. The metabolites and carotenogenesis genes involved in lutein biosynthesis were detected. This study would have substantial implications for natural lutein production by *C. zofingiensis* mutant LUT-4.

## 2. Results and Discussion

### 2.1. Isolation and Pigment of C. zofingiensis LUT-4 Strain

**Figure 1:**
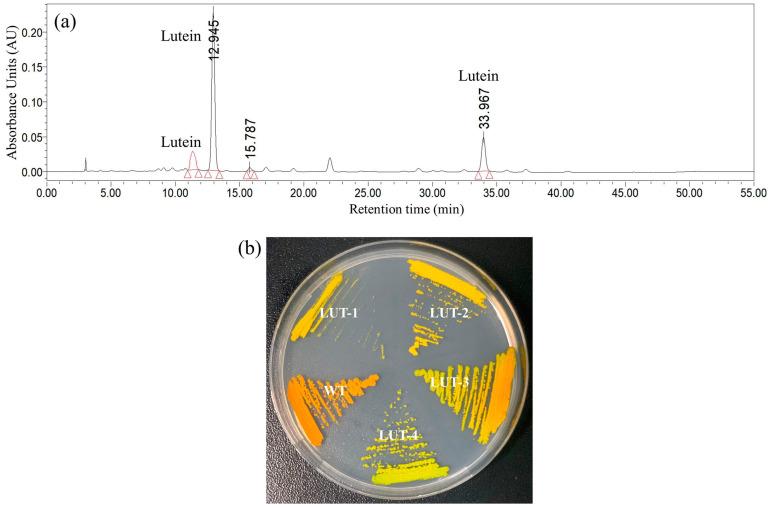
The comparison between wild-type (WT) and LUT-4. (**a**) The pigment profiles of lutein colonies were analyzed by using HPLC. (**b**) Color appearance of *C. zofingiensis* wild-type and lutein colonies on agar plates under heterotrophic conditions.

From the ethyl methyl sulfonate (EMS) mutagenesis pool, the colonies that are differentiated from wild-type (WT) by color were chosen to screen the mutants that can synthesize lutein, other than astaxanthin. A total of 28 mutants with a yellow or yellow-green appearance in color were identified from 30,000 mutants. Through pigment analysis by using HPLC, four stable mutants (LUT-1, LUT-2, LUT-3, and LUT-4) were selected due to their capacity to accumulate lutein (Figure 1a). In contrast to the wild-type, these mutants accumulated a large quantity of lutein but lacked astaxanthin. When cultivated as colonies on agar under heterotrophic conditions, they exhibited a yellow or yellow–green color, while the wild type was an orange color (Figure 1b).

**Figure 2:**
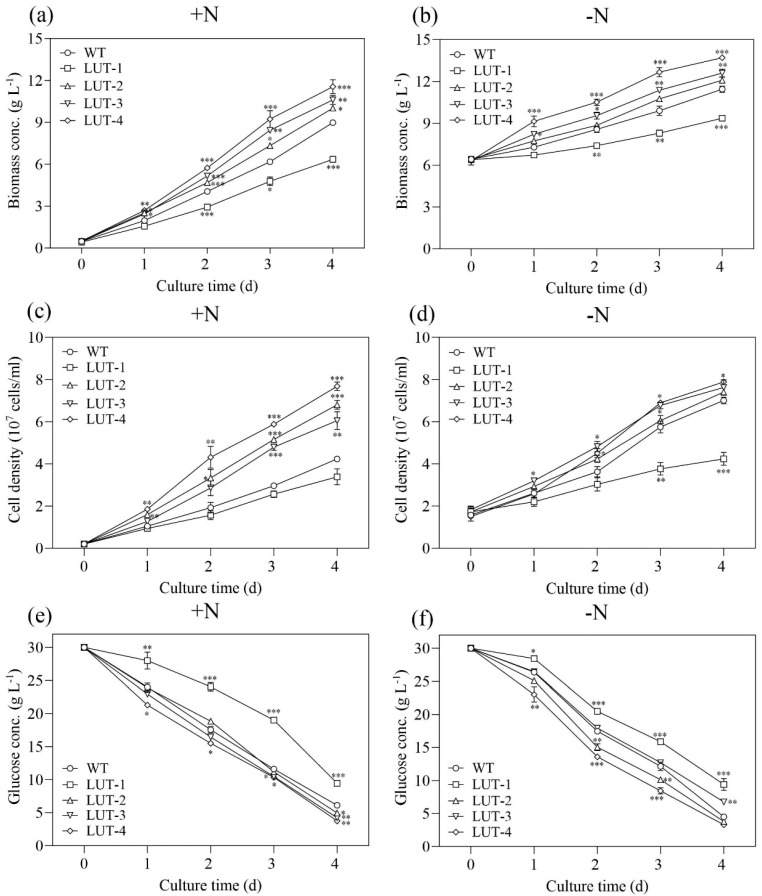
The growth and glucose consumption of wild-type (WT) and lutein mutants of *C. zofingiensis* under N-replete and N-deprived conditions. (**a**,**b**) Biomass concentration. (**c**,**d**) Cell density. (**e**,**f**) Glucose concentration. Values represent mean ± SD (*n* = 3). *, **, and *** are statistically significant at *p* < 0.05, *p* < 0.01, and *p* < 0.001, while lutein mutants are compared, respectively, with WT, at a given time.

When microalgal cells were cultivated under heterotrophic conditions, the biomass concentration and cell density of lutein mutants exhibited significant differences from that of WT (Figure 2a,b). As compared to WT and other mutants, LUT-4 showed significantly increased biomass accumulation, up to the highest yield of 11.55 g·L^−1^ and 13.7 g·L^−1^ on Day 4 under N-replete and N-deprived conditions, demonstrating 29.3% and 19.8% higher yield than that of WT (8.93 g·L^−1^, 11.44 g·L^−1^, *p* < 0.001), respectively. Furthermore, the biomass yields of LUT-2 and LUT-3 were also higher compared with WT under heterotrophic conditions (Figure 2a,b). Conversely, the biomass yield of LUT-1 was significantly lower compared to WT (*p* < 0.001, Figure 2a,b). LUT-2, LUT-3, and LUT-4 cells were dividing faster than those of WT under heterotrophic conditions. On Day 4, under N-replete conditions, the cell density of LUT-2, LUT-3, and LUT-4 was 6.8 × 10^7^ cell·mL^−1^, 6.05 × 10^7^ cell·mL^−1^, and 7.68 × 10^7^ cell·mL^−1^, which was 1.6-, 1.4-, and 1.8-fold higher than that of WT (4.23 × 10^7^ cell·mL^−1^), respectively. Under N-deprived conditions, the cell density of LUT-2, LUT-3, and LUT-4 was 7.41 × 10^7^ cell·mL^−1^, 7.62 × 10^7^ cell·mL^−1^, and 7.89 × 10^7^ cell·mL^−1^, respectively, on Day 4, which was higher compared to WT (7.01 × 10^7^ cell·mL^−1^, Figure 2d).

A prevalent organic carbon source utilized in the heterotrophic cultivation of microalgae is glucose. The glucose consumption of lutein mutants under N-replete and N-deprived conditions is presented in Figure 2c,d. The results showed that the glucose consumption of lutein mutants differed from WT. After four days of cultivation, the residual glucose concentration of LUT 1, LUT2, LUT-3, and LUT-4 under N-replete conditions was 9.45 g·L^−1^, 4.95 g·L^−1^, 4.22 g·L^−1^, and 3.75 g·L^−1^, respectively (Figure 2e). The utilization of glucose in LUT-2, LUT-3, and LUT-4 was quicker compared to WT under N-replete conditions. Similarly, the residual glucose concentration of LUT-2 and LUT-4 in the culture medium under N-deprived conditions was utilized quicker compared with WT (Figure 2f). Conversely, the glucose consumption of LUT-1 and LUT-3 was slower than that of WT.

**Figure 3:**
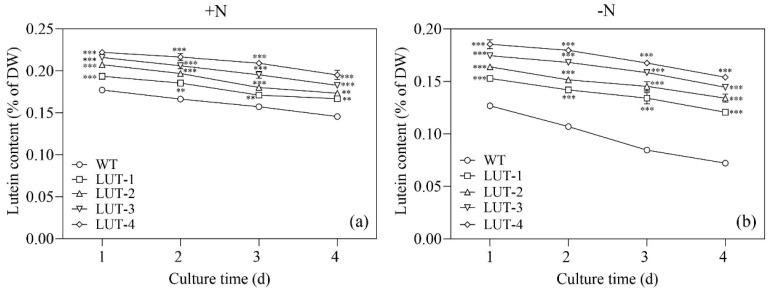
Changes in lutein contents in lutein mutants of *C. zofingiensis* strain under N-replete (**a**) and N-deprived (**b**) conditions. Values represent mean ± SD (*n* = 3). ** and *** are statistically significant at *p* < 0.01 and *p* < 0.001, while lutein mutants are compared with WT, respectively, at a given time.

HPLC analysis demonstrated that LUT-1, LUT-2, LUT-3, and LUT-4 mainly accumulated lutein. The content of lutein in LUT-1, LUT-2, LUT-3, and LUT-4 under N-replete conditions was 0.167%, 0.173%, 0.183%, and 0.195% of DW on Day 4, which was 14.4%, 18.5%, 25.3%, and 33.6% higher compared with WT (0.146% of DW), respectively (Figure 3a). Under N-deprived conditions, the lutein content of LUT-1, LUT-2, LUT-3, and LUT-4 reached 0.127%, 0.134%, 0.144%, and 0.154% of DW on Day 4, respectively, which was significantly higher compared with WT (0.072% of DW, *p* < 0.001, Figure 3b).

Collectively, LUT-4 exhibited increased cellular mass, quicker cell division, and high lutein content under heterotrophic conditions in comparison to WT. The biomass yield of LUT-4 was significantly higher compared with WT under heterotrophic conditions, suggesting that the specific mutations did not impact the growth potential. In general, mutagenesis is defined as the process by which heritable alterations arise in the genetic information of an organism [19]. EMS was classified as a chemical mutagen due to its ability to induce insertion and site-direction mutagenesis in DNA sequences [20]. The mutations could occur not only in genes related to carotenoid synthesis but also in other genes. In addition, the mutation sites need to be detected in further studies.

### 2.2. Effect of Light Intensity on Growth and Lutein Accumulation of LUT-4

**Figure 4:**
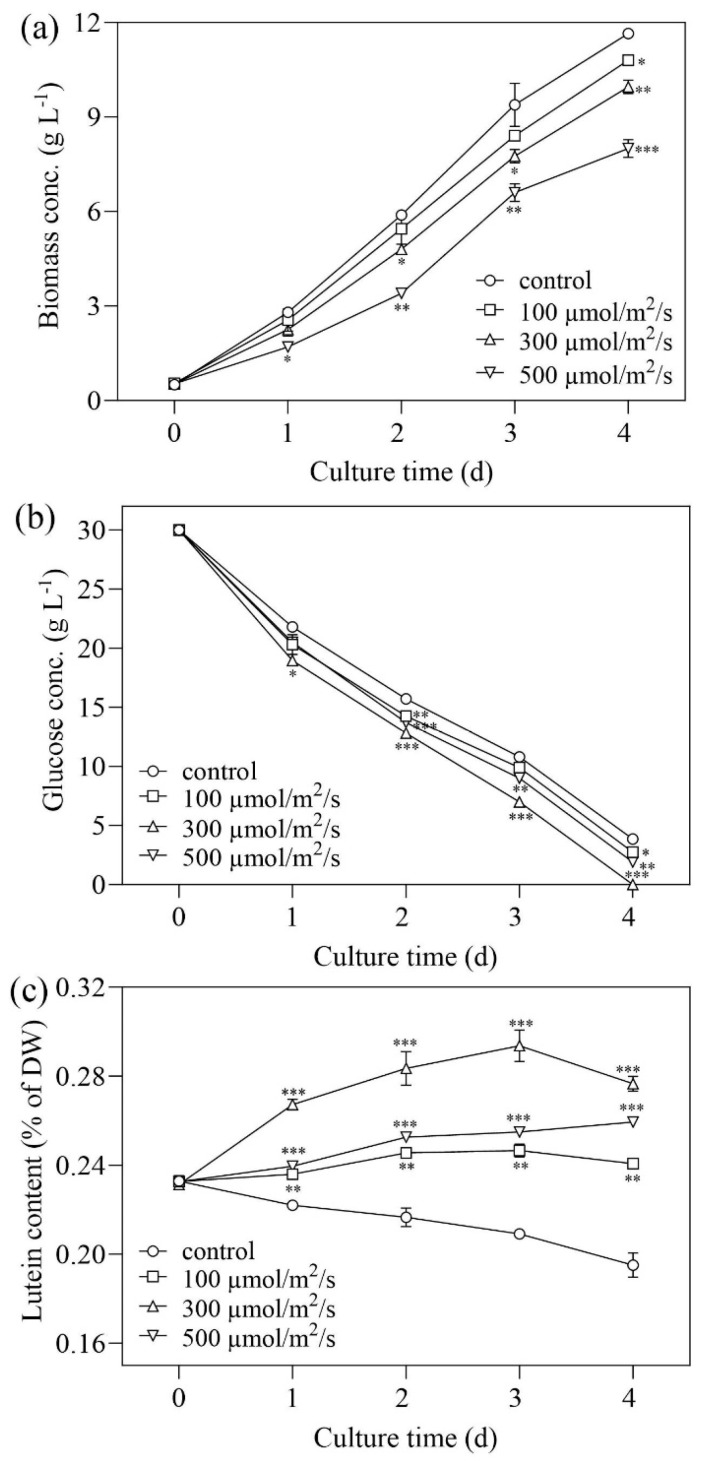
Effects of light intensities on the growth of *C. zofingiensis* mutant LUT-4 and lutein accumulation. (**a**) Biomass concentration. (**b**) Glucose concentration. (**c**) Lutein content. Values represent mean ± SD (*n* = 3). *, **, and *** are statistically significant at *p* < 0.05, *p* < 0.01, and *p* < 0.001, while 100, 300, 500 µmol/m^2^/s light intensity are compared, respectively, with the control, at a given time.

When LUT-4 was cultivated heterotrophically, the lutein content declined to 0.154% of DW. In contrast to the outcomes observed in comparable research, this performance remains unsatisfactory [21,22]. Light intensity is frequently the most influential factor in cell growth and lutein accumulation [23]. An increase in light intensity from 100 µmol/m^2^/s to 500 µmol/m^2^/s resulted in a substantial decrease in biomass concentration (*p* < 0.05, Figure 4a). After four days of cultivation, the highest biomass obtained under 100 µmol/m^2^/s, 300 µmol/m^2^/s, and 500 µmol/m^2^/s was 10.8 g·L^−1^, 9.95 g·L^−1^, and 8 g·L^−1^, which was 7.3%, 14.6%, and 31.3% lower, respectively, compared with the control (11.65 g·L^−1^). When the light intensity was increased from 100 µmol/m^2^/s to 500 µmol/m^2^/s, the glucose consumption was enhanced (Figure 4b).

Furthermore, the composition of microalgae cells may also alter in response to variations in ambient light intensity, particularly for light-related chemicals like carotenoids and chlorophyll [11]. The research discovered that under high light intensity, the quality of main xanthophylls in microalgae, such as lutein, tended to decrease [24]. The reduction in size of light-harvesting cells, where lutein is located and generated, may be the reason for the drop in lutein content under high-intensity light [4]. As shown in Figure 4c, the highest lutein content reached 0.294% of dry weight (DW) when a light intensity at 300 µmol/m^2^/s was used. When the light intensity was increased, the lutein content decreased. Under 100 µmol/m^2^/s, the lutein content reached 0.26% of DW on Day 4, which was slightly lower than that under 300 µmol/m^2^/s (0.277% of DW). Among the results above, the light intensity of 300 µmol/m^2^/s was optimal for lutein production, and the highest yield of lutein was 0.028 g·L^−1^, on Day 4.

The results found above suggest that light intensity has inverse effects on biomass and lutein accumulation. For instance, the microalgae cultivated under 300 µmol/m^2^/s had a high lutein concentration, but their biomass yield was slightly lower compared to the control. Notably, the lutein content achieved in this study was better than most of those reported in the literatures [22,25]. LUT-4 appeared to be an excellent microalgal feedstock for the commercial production of lutein, as demonstrated by the present study, which attributes its lutein content to a comparatively high level of accumulation at a light intensity of 300 µmol/m^2^/s. To further increase the lutein yield of this strain on a large scale and thereby render it more economically viable, additional engineering research is required.

### 2.3. Effects of Light Intensity on Biochemical Composition

**Figure 5:**
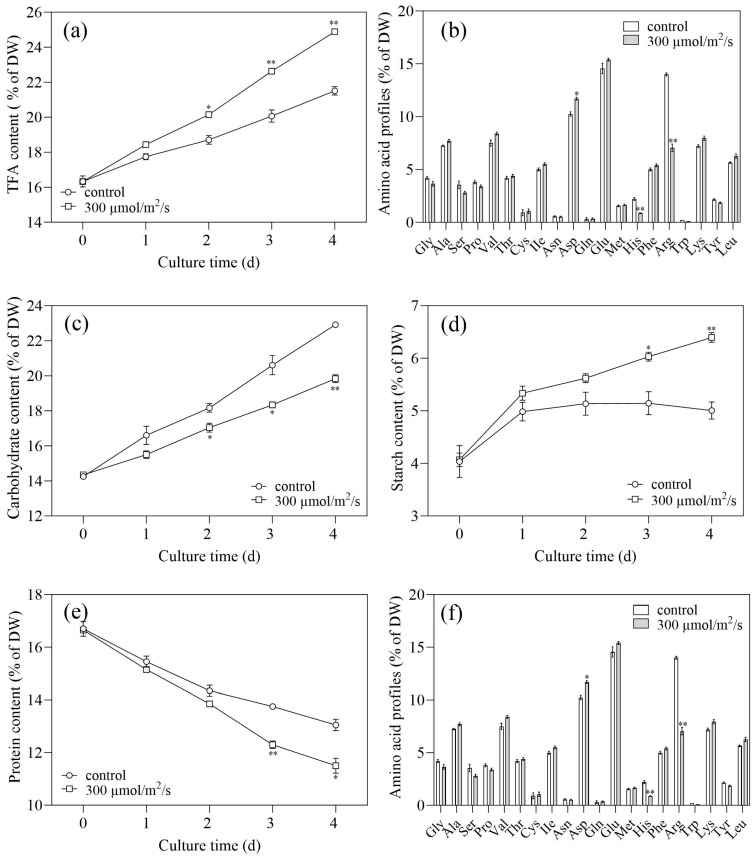
The biochemical changes in *C. zofingiensis* mutant LUT-4 were affected by optimal light intensity. (**a**) TFA contents. (**b**) Fatty acid profiles. (**c**) Carbohydrate content. (**d**) Starch content. (**e**) protein content. (**f**) Amino acid profiles. Values represent mean ± SD (*n* = 3). * and ** are statistically significant at *p* < 0.05 and *p* < 0.01, while 300 µmol/m^2^/s light intensity is compared with the control, at a given time.

Based on the results above, 300 µmol/m^2^/s was the optimal light intensity for lutein accumulation in LUT-4. To make better use of this cultivation mode, the biological profiles were investigated in the following experiment. Under 300 µmol/m^2^/s, the TFA content in LUT-4 rose from 16.32% to 24.89% of DW, which was 15.7% higher compared with the control (21.51% of DW, Figure 5a). On Day 4, when LUT-4 was grown at a light intensity of 300 µmol/m^2^/s, the main fatty acids were C16:0, C18:1, C18:2, and C18:3 (Figure 5b). The abundances of C16:0 and C18:3 declined under 300 µmol/m^2^/s. In contrast, the abundance of C18:1, which comprised as much as 32.55% of TFA, increased significantly, while C18:3 decreased drastically (Figure 5b). The results confirmed the previous research, which discovered that *C. zofingiensis* increased the abundance of C18:1 and reduced the abundances of C18:3 in response to stressful conditions [26].

As shown in Figure 5e, the light intensity of 300 µmol/m^2^/s significantly decreased protein content, by 11.5% of DW. In several reported microalgae, intracellular protein tended to degrade under stress conditions, providing the carbon skeleton and energy for lipid biosynthesis [27]. The degraded protein was suggested to first guide carbohydrate biosynthesis and then lipid. The results showed that the content of carbohydrates was significantly lower compared to the control (*p* < 0.05). However, the starch content in LUT-4 under 300 µmol/m^2^/s increased from 4.07% to 6.4% of DW.

The results showed that the amino acid composition of LUT-4 was the same as the wild-type [28]. Interestingly, light intensity altered the composition of essential amino acids including Gly, Ala, Ser, Pro, Val, Thr, IIe, Asp, Glu, His, Phe, Arg, Lys, Tyr, and Leu (Figure 5f). Notably, the contents of His and Arg dramatically reduced (*p* < 0.01). Moreover, the contents of Ala, Val, Thr, IIe, Asp, and Glu increased in LUT-4. Thus, it could be concluded that light intensity altered the protein composition of LUT-4. Taken together, the alterations in biochemical compositions under 300 µmol/m^2^/s were mainly caused by the utilization of organic carbon.

### 2.4. Carbon Availability Comparison

**Figure 6:**
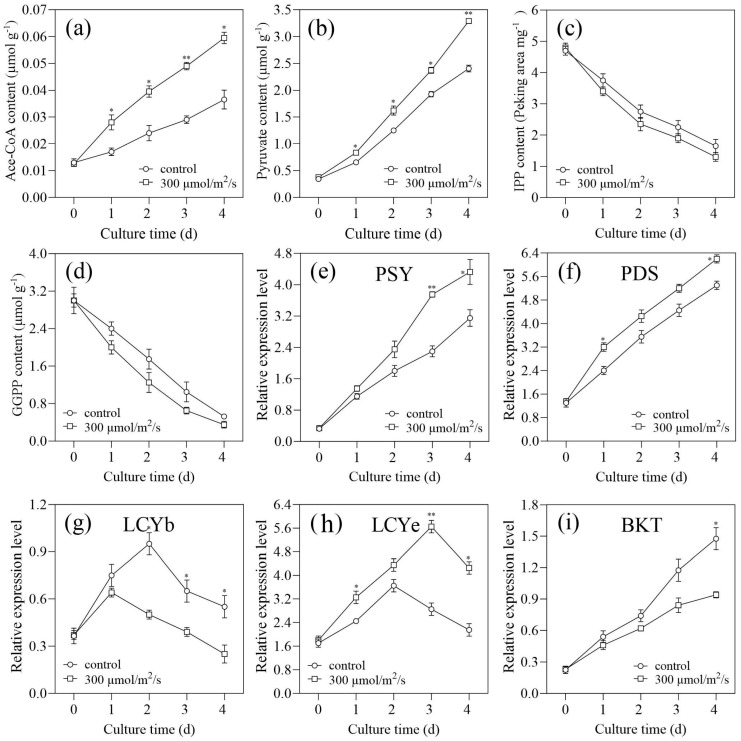
Effects of 300 µmol/m^2^/s light intensity on metabolism and gene expression of *C. zofingiensis* mutant LUT-4. Variations in (**a**) Ace-CoA, (**b**) pyruvate, (**c**) isopentenyl pyrophosphate (IPP), and (**d**) geranylgeranyl diphosphate (GGPP) contents during cultivation periods. Variation in gene expression in (**e**) phytoene synthase (*PSY*), (**f**) phytoene desaturase (*PDS*), (**g**) Lycopene beta cyclase (*LCYb*), (**h**) lycopene epsilon cyclase (*LCYe*), and (**i**) Beta-carotenoid ketolase (*BKT*) during cultivation periods. Values represent mean ± SD (*n* = 3). * and ** are statistically significant at *p* < 0.05 and *p* < 0.01, while 300 µmol/m^2^/s light intensity is compared with the control, at a given time.

The activity of the synthetic pathway and the availability of carbon molecules are both critical for lipid biosynthesis [29]. As shown in Figure 6a, Ace-CoA content increased significantly at a light intensity of 300 µmol/m^2^/s, which was primarily via the central carbon metabolism (*p* < 0.05). Ace-CoA serves as the primary precursor for lipid biosynthesis and may undergo conversion into C16:0 and C18:0 as part of fatty acid metabolism [30]. The lipid content is determined by the activity of the synthetic pathway when the precursor is adequate (i.e., Ace-CoA). Generally, Ace-CoA could join the biosynthesis of fatty acids continuously and rapidly. The results showed that the light intensity of 300 µmol/m^2^/s could increase Ace-CoA content and activate the pathway for fatty acid biosynthesis. These findings were consistent with the results above. Briefly, the C16:0 content was reduced and the C18:1 content was increased at a light intensity of 300 µmol/m^2^/s (Figure 5b).

Glucose can be directed to participate in carotenoid metabolism once it has been assimilated by cells [31]. In this study, the lutein content in LUT-4 could be increased to 0.294% of DW at a light intensity of 300 µmol/m^2^/s. The synthesis of carotenoids in green alga commences with IPP, which is generated through the non-mevalonate pathway by 3-phosphoglyceraldehyde (G3P) and pyruvate [32]. The light intensity of 300 µmol/m^2^/s significantly improved pyruvate content compared to that of the control, which suggested this cultivation method supplied more available carbon molecules for carotenoid synthesis (*p* < 0.05, Figure 6b). However, the content of IPP and GGPP (i.e., the downstream metabolite of IPP) declined at a light intensity of 300 µmol/m^2^/s (Figure 6c,d). In comparison to the control, the lutein content was greater, even though the GGPP content was lower at a light intensity of 300 µmol/m^2^/s. The findings indicated that, with the aid of suitable light intensity, carbon molecules could be converted to lutein, resulting in a reduction in its precursor metabolites. Thus, lutein accumulation was dependent on the availability of an abundance of carbon molecules, which was demonstrated by the carbon-use nature of lutein synthesis. In addition, the light intensity of 300 µmol/m^2^/s resulted in the highest lutein content of 0.294% of DW, on Day 3 (Figure 4c). The implemented strategy (i.e., at a light intensity of 300 µmol/m^2^/s) increased carbon availability relative to the control by increasing the rate of glucose uptake and pyruvate content. The consumption rate of GGPP content was accelerated by the strategy. As previous investigations have unveiled, the conversion of GGPP involves a limited number of enzymes, which are likewise examined in the subsequent section [27,33].

Several essential enzymes, including *PSY*, *PDS*, *LCYb*, *LCYe*, and *BKT*, are typically involved in sequential chain transformations that generate the diverse carotenoid family [34]. The carotenoid metabolism gene *PSY* limits the pace at which two GGPP contents may be condensed to produce lycopene [32]. The *PDS* gene, which is involved in carotenoid biosynthesis, enables microalgae to convert phytoene into ζ-carotene [35]. Compared with the control, the light intensity of 300 µmol/m^2^/s significantly improved the relative expression level of *PSY* and *PDS*, respectively (*p* < 0.05, Figure 6e,f). *LCYe* is the gene responsible for lutein synthesis, whereas *LCYb* and *BKT* are genes intimately associated with astaxanthin synthesis [27,36]. Alterations in these genes may direct the flow of carbon into carotenoids, either as primary or secondary metabolites [36]. LUT-4 cells cultivated under 300 µmol/m^2^/s increased the relative expression level of *LCYe*, which suggested the strategies had the potential to enhance the synthetic ability of Lutein (*p* < 0.05, Figure 6h). Moreover, the light intensity (i.e.,300 µmol/m^2^/s) significantly decreased the expression level of *LCYb* and *BKT* compared to that under the control (*p* < 0.05). Thus, the light intensity of 300 µmol/m^2^/s had the most pronounced impact on promoting lutein synthesis, aligning with the observed lutein content values. The current study revealed that LUT-4 seems to be a great microalgal feedstock for commercial lutein production because of its comparatively high lutein content at a light intensity of 300 µmol/m^2^/s.

## 3. Materials and Methods

### 3.1. Microalgal Strains and Growth Conditions

The American Type Culture Collection (ATCC, Rockville, MD, USA) provided the wild-type *Chromochloris zofingiensis* (ATCC30412), which was cultivated in the modified Endo medium. The pH of the modified Endo medium was first set to 6.5 by using the 3 M NaOH solution [37]. After transferring a single colony of *C. zofingiensis* into 250 mL Erlenmeyer flasks with 100 mL sterilized medium, the flasks were orbitally shaken at 180 rpm for seven days at 26 °C.

Mutagenesis was performed by harvesting WT cells at the early logarithmic phase by using centrifugation (1000, 5 min) and washing twice with phosphate-buffered saline (PBS, pH = 6.5). After treating microalgal cells for one hour in the dark with 2% (*w*/*v*) ethyl methyl sulfonate (EMS, Sigma-Aldrich, St. Louis, MO, USA), 10% (*w*/*v*) Na_2_SO_3_ was added to stop the mutagenesis process. The treated cells were resuspended in the refreshed modified Endo medium for 24 h in the absence of light after being rinsed and washed with PBS. Agar plates with modified Endo medium were used to cultivate approximately 20,000 colonies after mutagenesis. In contrast to the orange WT colonies, those exhibiting a green–yellow hue were chosen for cultivation in liquid-modified Endo medium supplemented with 30 g·L^−1^ glucose.

Log phase microalgal cells were inoculated into N-replete-modified Endo medium at an initial cell density of 2 × 10^6^ cell·mL^−1^ for 4 days of heterotrophic cultivation. The gathered cells were then reintroduced into N-deprived-modified Endo medium, while the initial biomass concentration was kept at 6 g·L^−1^. Furthermore, various light intensities (i.e., 100, 300, and 500 µmol/m^2^/s) were applied to optimize the light supply conditions to increase lutein production by LUT-4. The same sample of microalgal cells was inoculated for the following experiment.

### 3.2. Determination of Dry Weight and Residual Glucose Concentration

Samples were taken at each time point, cleaned twice with 0.5 M NH_4_HCO_3_, and passed through a 1.2 μm pore-size dry GF/C filter paper (Whatman, Life Sciences, Maidstone, UK), which had been pre-weighed and dried at 105 °C in an oven for a whole night. Before measuring the dry weight, the Whatman GF/C filter paper was put in a desiccator for 20 min to enable the temperature to drop. The measurement of glucose concentration was performed by utilizing a Gold-Accu Glucose Monitoring System (Model BGMS-1; Sinocare Inc., Changsha, China).

### 3.3. Lutein Content Determination

Samples were extracted by utilizing previously published procedures [8]. A YMC Carotenoid (250 × 4.6 mm, 5 μm) column separated the lutein at 30 °C. A sample aliquot of ten microliters was introduced into the Waters Associates, Milford, MA, USA HPLC system outfitted with a 2998 photodiode array detector (Waters, Milford, MA, USA). Eluent A (methanol/methyl tert-butyl ether/water = 81:15:4, *v*/*v*) and eluent B (methanol/methyl tert-butyl ether/water = 43.5:52.5:4, *v*/*v*) made up the mobile phase. A gradient procedure was employed to separate the lutein: 0% B for 45 min, followed by a two-minute increase in gradient to 100% of B, and an eight-minute hold at 0% B. The rate of flow was 1.0 mL·min^−1^. For quantification, the lutein standard (Sigma-Aldrich, St. Louis, MO, USA) was utilized as the calibrant. Lutein was detected by comparing the retention durations and absorption spectra of specific peaks in the chromatogram to the standards, and peak areas were extracted for quantification using calibrant curves produced with the standards.

### 3.4. Determination of Organic Composition

Methods previously documented were employed to extract the samples [12]. The quantification of total fatty acid content was accomplished through the combination of Agilent 7890B gas chromatography and 5977A mass spectrometry (GC-MS) (Agilent, Santa Clara, CA, USA).

The supernatant was discarded after the samples were ground and incubated for 30 min at 80 °C with an 80% ethanol solution. Amylase was introduced into the granules to aid in the hydrolysis of starch, which was accomplished by heating the pellets. Anthrone and sulfuric acid were added to the released glucose and incubated for 10 min at 95 °C. The starch content was determined by translating the optical density at 562 nm to units of glucose.

Samples were incubated with 0.5 mL of acetic acid for 20 min at 80 °C before being treated with 10 mL of acetone. After the supernatant was removed, 2.5 mL of 4 M trifluoroacetic acid was added and the samples were then heated for 4 h. The optical density at 490 nm (OD_490_) was determined by boiling samples in a solution containing chromogenic reagent for twenty minutes. A standard curve was constructed by utilizing glucose to determine the total content of carbohydrates.

The protein content was determined in strict adherence to the established approach [38]. The protein content was calculated by using a conversion factor of 6.25 to the total nitrogen content of the samples, as measured by an automated Kjeldahl analyzer (UDK 159-VELP, Usmate Velate, Italy). The amino acid composition was analyzed by using the A300 auto amino acid analyzer (Membra Pure, Bodenheim, Germany) equipped with a TS263 column.

### 3.5. Isopentenyl Pyrophosphate (IPP) and Geranylgeranyl Diphosphate (GGPP) Quantification

To ascertain IPP and GGPP, microalgal cells were extracted and subsequently subjected to liquid nitrogen pulverizing to disrupt them. A vacuum freeze-drying machine (LP 20, IIshinbiobase Co., Ltd., Dongducheon, Korea) was then utilized to dehydrate the metabolites that had been extracted with methanol. The samples were purified by a solid-phase extraction (SPE) column (Waters, Milford, MA, USA) and analyzed by UPLC-MS/MS (Waters, Milford, MA, USA), according to the earlier study [39]. The standards of IPP and GGPP were acquired from Sigma-Aldrich (St. Louis, MO, USA).

### 3.6. Measurement of Ace-CoA and Pyruvate

For measurement of Ace-CoA contents, the microalgal cells were extracted and analyzed by using the Ace-CoA assay reagent (Sigma, MAK039). Pyruvate content was determined by extracting pyruvate and quantifying it using the pyruvate assay reagent (Sigma, MAK071), in accordance with the provided instructions.

### 3.7. Determination of Expression Levels of mRNA

The RNA Plant Plus Reagent (Tiangen, Beijing, China) was utilized to extract the total RNA of *C. zofingiensis*, in accordance with the manufacturer’s instructions. Reverse transcription of the RNA to cDNA was then performed according to the instructions, using the QuantScript RT Kit reagent (Tiangen, Beijing, China). One Step SYBR PrimeScript PLUS RT-PCR Kit reagent (TaKaRa, Tokyo, Japan) was utilized to conduct real-time PCR. Table 1 lists the available Primers. The mRNA expression level was stabilized by using the *C. zofingiensis* actin (ACT) gene as the internal control.

### 3.8. Statistical Analysis

The experiments in this research were carried out in triplicate. The statistical differences between the two data sets were determined by using the unpaired Student’s *t*-test. A combination of one-way ANOVA and Dunnett’s post hoc test was utilized to assess multiple comparisons. The statistical analysis was conducted by using GraphPad Prism version 10 (GraphPad Software, La Jolla, CA, USA). The software allowed for approval at a level below *p* < 0.05.

## 4. Conclusions

In this study, four lutein mutants were isolated from chemical mutagenesis. By comparing the growth and lutein content, LUT-4 was selected for further study. Results showed that 300 µmol/m^2^/s was the most suitable light intensity for lutein accumulation. The biochemical composition, Ace-CoA, pyruvate, IPP, and GGPP content alterations demonstrated that the light intensity could enhance the use of organic carbon for lutein biosynthesis. Moreover, the elevated expression of *PSY*, *PDS*, and *LCYe* genes could facilitate the generation of lutein. Overall, this study supplied a feasible technique for producing natural lutein by LUT-4.

## Figures and Tables

**Table 1:** PCR primers used for RT-PCR to quantify expression level of carotenogenesis genes.

Gene	Forward (5′-3′)	Reverse (5′-3′)
*PSY*	CACCAGGTTGTCAGAGTCCA	ACTAGTGTGTTGCTGACTCT
*PDS*	GATGAATGTATTTGCTGAACT	GGCCAGTGCCTTAGCCATAG
*LCYe*	TCAAAGCACAGGCGAACAAACA	AACGTCGGGACCTATAAGTCCG
*LCYb*	CGCAGGCGAAAAATTCCTGT	TAAGGAATGTCACACCGCTGG
*BKT*	GGTGCTCAAAGTGGGGTGGT	CCATTTCCCACATATTGCACCT
*ACT*	TGCCGAGCGTGAAATTGTGA	CGTGAATGCCAGCAGCCTCCA

## Data Availability

Data are contained within the article.
